# A complex role of chromogranin A and its peptides in inflammation, autoimmunity, and infections

**DOI:** 10.3389/fimmu.2025.1567874

**Published:** 2025-04-30

**Authors:** Maciej Maj, Karolina Hernik, Kaja Tyszkiewicz, Maja Owe-Larsson, Alicja Sztokfisz-Ignasiak, Jacek Malejczyk, Izabela Janiuk

**Affiliations:** ^1^ Department of Histology and Embryology, Center of Biostructure Research, Medical University of Warsaw, Warsaw, Poland; ^2^ Institute of Health Sciences, Faculty of Medical and Health Sciences, University of Siedlce, Siedlce, Poland

**Keywords:** autoimmune, catestatin (CST), chromofungin (CHR), chromogranin A (CgA), inflammation, pancreastatin (PST), vasostatin (VS), WE-14

## Abstract

Chromogranin A (CgA), mostly known as a nonspecific neuroendocrine tumor marker, was the first glycoprotein from the granin family characterized as a prohormone for various bioactive peptides including vasostatin I/II (VS-I, VS-II), catestatin (CST), chromofungin (CHR), pancreastatin (PST), WE-14, and others. CgA and its derivatives present various functions, often antagonistic, in maintaining body homeostasis and influencing the immune system. This review aims to summarize the not fully understood role of CgA and its derivatives in inflammation, autoimmunity, and infections. CgA seems to be involved in the complex pathophysiology of cardiovascular disorders, neurodegenerative diseases, and other conditions where immune system dysfunction plays a role in the onset and development of the disease (e.g. systemic lupus erythematosus (SLE), inflammatory bowel disease (IBD), or rheumatoid arthritis (RA)). However, the direct immunomodulatory role of CgA is difficult to assess since many of its activities may be linked with its peptides. CST and VS-I are considered anti-inflammatory molecules, due to M2 macrophage polarization stimulation and downregulation of certain proinflammatory cytokines. Conversely, PST is reported to stimulate proinflammatory M1 macrophage polarization and Th1 lymphocyte response. Thus, the final effects of CgA in inflammation may depend on its cleavage pattern. Additionally, peptides like CST, VS-I, or CHR exert direct antimicrobial/antifungal activities. CgA, WE-14, and other less-known CgA-derived peptides have also been reported to trigger autoimmune responses, highly studied in type 1 diabetes mellitus. Overall, CgA and its derivatives have an interesting but complex role in immunity, however, their specific roles require further research.

## 1 Introduction

**Figure 1:**
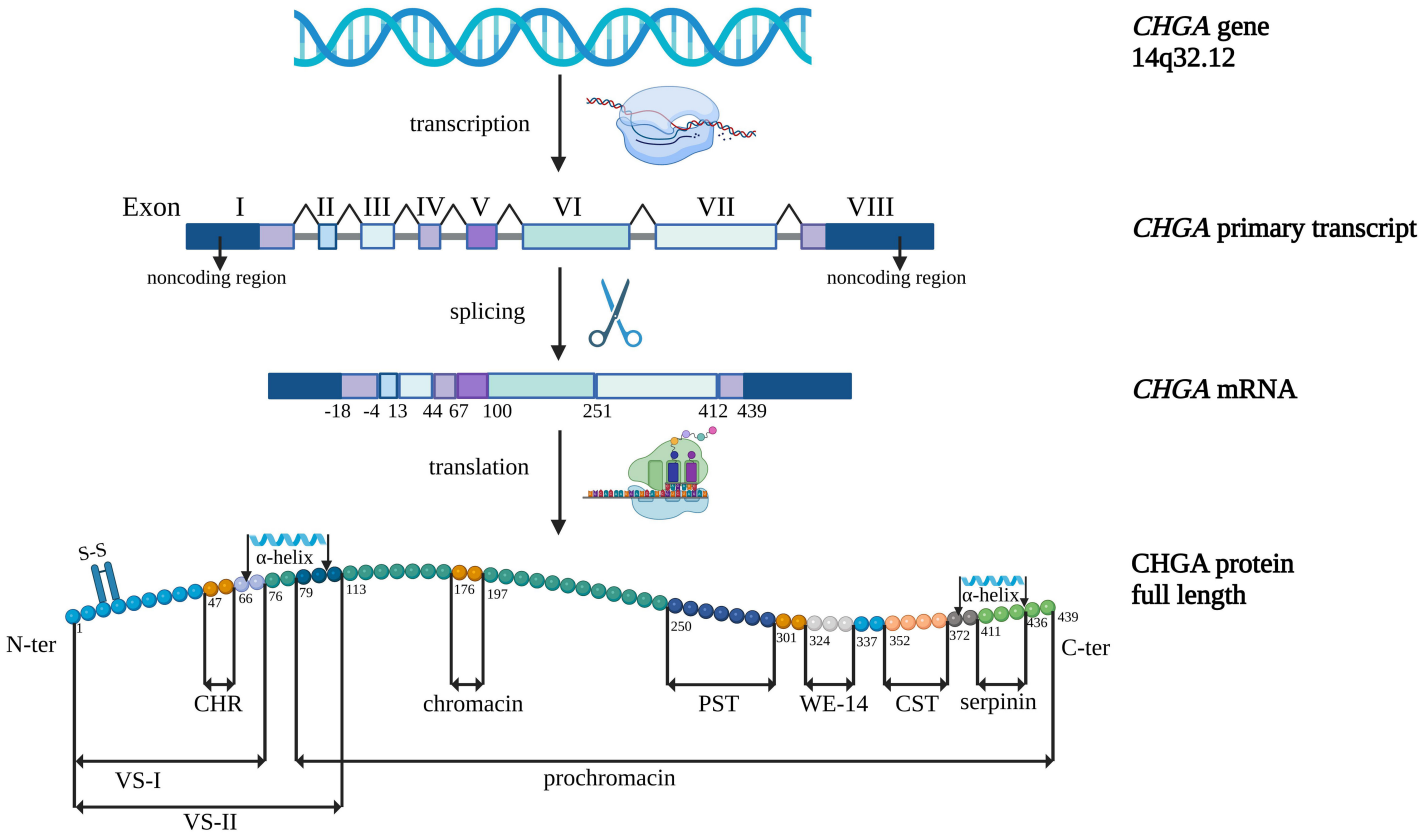
Structure of the human chromogranin A gene (*CHGA*) and *CHGA-*encoded peptides. CHR, chromofungin; CST, catestatin; PST, pancreastatin; VS-I, vasostatin I; VS-II, vasostatin II. Created with BioRender.com/c35u482.

Granins are secretory proteins localized in the cell cytoplasmic electron-dense granules (1). A major member of the granin family is chromogranin A (CgA), an acidic hydrophilic glycoprotein composed of 439 amino acids in humans with a molecular mass of ~49 kDa. Primarily CgA was identified in the chromaffin granules of the bovine adrenal medulla (2–6). The structure of the human CgA gene (CHGA) and CHGA-encoded peptides are shown in **Figure 1**.

The human CHGA gene is located on chromosome 14 (14q32.12) (7, 8) and consists of eight exons separated by seven intronic sequences (9–11). CgA contains a signal peptide containing 18 amino acids, a 5’-untranslated region (5’UTR), and a 3’-untranslated region (3’UTR). The 5’UTR region with most of the signal peptide is encoded by exon I, while the C-terminal fragment of the protein and the 3’UTR region are encoded by exon VIII (9). Exons II-V encode vasostatins (VS-I, VS-II) and exon III encodes cysteine residues necessary to form the disulfide loop of CgA. The most bioactive peptides such as catestatin (CST), pancreastatin (PST), and WE-14 are encoded by exon VII, whereas serpinin is encoded by exons VII and VIII (4).

The structure of CgA contains 8–10 dibasic sites (4, 12, 13), which are potential sites of proteolytic processing by endogenous proteases such as prohormone convertase PC1 and PC2 (14), carboxypeptidase H (15), cathepsin-L (16), plasmin (17, 18), thrombin (19) or other exogenous proteases located both on the outside of the cell membrane and present in the plasma (20).

CgA was the first granin characterized as a prohormone for various biologically active peptides. The most important and the best examined CgA derivatives are VS-I (1–76) (21), VS-II (1–113) (22, 23), chromofungin (CHR) (47–66) (24), chromacin (176–197) (25, 26), prochromacin (79–439) (26, 27), PST (250–301) (28), CST (352–372) (29), serpinin (411–436) (30, 31) and WE-14 (324–337) (32) (**Figure 1**).

The first CgA-derived polypeptide was identified in the glucose-stimulated porcine pancreas. It is called PST, and its main function is to inhibit insulin secretion (28). Since that discovery, numerous studies have been carried out in which it has been proven that CgA is a prohormone for many other polypeptides that exhibit a broad range of biological activity, including antimicrobial (33), pro- and anti-angiogenic effects (17, 19, 34). The function of CgA-derived peptides is also related to calcium and glucose homeostasis, endothelial permeability, myocardial contractility, innate immunity, and anti- and pro-adrenergic effects (17, 35, 36).

CgA is a unique molecule because peptides derived from its breakdown have antagonistic effects in maintaining homeostasis in the body (37, 38). Full-length CgA seems to inhibit angiogenesis, but it is also a precursor of pro-angiogenic (VS-II) (39) and anti-angiogenic (VS-I) peptides (19, 40). In turn, glucose homeostasis is maintained by PST, which is an anti-insulin peptide (41–43), and CST, which presents a pro-insulin effect (44, 45).

Considering that CgA and its cleavage products were found to be associated with various pathological conditions (such as cancer, autoimmune diseases, and cardiovascular disorders), the present review aims to summarize their putative role in infection, inflammation, and inflammatory diseases.

## 2 Chromogranin A

Apart from being a neuroendocrine tumor marker, CgA is elevated in several diseases. Blood circulating CgA was found to be elevated in cancer (4, 46), organ failure, inflammation, autoimmune diseases (e.g. Crohn’s disease (CD), diabetes mellitus type 1, giant cell arteritis, RA, SLE), renal failure, sepsis, primary hyperparathyroidism, and some cardiovascular diseases (e.g. atherosclerosis, heart failure, hypertension, Takayasu’s arteritis) (47, 48). It is worth noting that other factors such as pregnancy, exercise, or certain drugs like corticoids, and proton pump inhibitors may also contribute to elevated circulating CgA levels (49). In IBD, steroids and immunosuppressive thiopurine treatment tend to increase CgA circulating levels. Conversely, anti-TNF therapies and other biological treatments have been observed to reduce CgA levels significantly (50).

CgA seems to play a role in the complex pathophysiology of certain cardiovascular disorders (coronary artery disease, chronic heart failure), neurodegenerative diseases (such as Alzheimer’s disease) as well as in other conditions where the malfunction of the immune system contributes to the disease onset and progression like RA or IBD (49, 51, 52).

Elevated tissue and/or blood levels of CgA in diseases such as ulcerative colitis (UC) or prostate cancer can be partially explained because of an increased number of cells that secrete CgA, induced by cytokines such as IFN-γ, IL-1β, IL-6, or TNF (53–56). Animal models and *in vitro* research show that those cytokines may stimulate neuroendocrine differentiation in prostate cancer and prostatitis involving AMPK, gp130, and STAT3 signaling. Neuroendocrine differentiation has also been observed in non-small cell lung cancer, breast cancer, and gastric and colonic adenocarcinomas (56–61).

In inflammatory conditions, cytokines (IL-6, TNF) may also activate the hypothalamic-pituitary-adrenal axis leading to CgA release to the bloodstream along with catecholamines which are regulated by CgA (62, 63). CgA synthesis may also be upregulated by glucocorticoids (62).

Under pathological conditions, not only neuroendocrine cells express and secrete CgA. There is evidence that this glycoprotein is expressed along with B-type natriuretic peptide (BNP) in ventricles during heart failure (64). Interestingly, white blood cells may also be an important source of CgA - especially neutrophils, lymphocytes, and monocytes. Neutrophils stimulated by lipopolysaccharide (LPS) and LukE/D (from Staphylococcus aureus) revealed increased release of CgA and its shorter derivatives. This shows that CgA, either directly or via its cleavage products, plays a role in inflammatory and stress conditions (1, 62, 65).

Circulating CgA levels correlate with serum inflammatory markers like C-reactive protein (CRP), procalcitonin, IL-1β, IL-6, IL-8/CXCL8, IL-17C, or TNF in conditions such as gastritis, IBD, chronic heart failure or systemic inflammatory response syndrome. CgA and IL-17C are co-expressed in intestinal neuroendocrine cells in IBD (52, 62, 66–68).

Based on human UC biopsy sample examination and an animal model of colitis (induced by dextran sulfate sodium - DSS in CHGA gene knockout mice), a positive correlation between CgA and M1 macrophage-associated pro-inflammatory cytokines such as IL-1β, IL-6, and TNF have been observed. CgA expression also correlated negatively with M2 macrophage markers such as C-MYC, MR, CD1b, and IL-10. M2 macrophages, often called alternatively activated macrophages, mediate anti-inflammatory reactions and healing processes, leading to increased angiogenesis and collagen production via mediators such as IL-10, TGF-β, VEGF, and arginase. Thus, a modulatory role of CgA on M1/M2 macrophages probably leads to a progression of inflammation in UC and attenuates regeneration processes (69).

In an animal model of chronic kidney disease, 5/6th partial nephrectomy mice, knockout of the CHGA gene led to a significantly lower level of renal fibrosis. In the same study, mouse mesangial kidney cells incubated with CgA were reported to induce the release of Nitric Oxide (NO) as well as certain chemokines like CXCL1,2,5 and CCL2, 20 via the TLR 4/SR-A (type A scavenger receptor) pathway (63). CgA also positively correlated with increased chemotactic IL-8 and IL-18 production during intestinal inflammation (70).

More and more data support the hypothesis that CgA is an important stimulator of neuroinflammation. CgA is a known stimulator of microglia, leading to higher expression, maturation, and secretion of pro-inflammatory cytokines such as IL-1β, IL-18, and TNF, possibly via activation of nuclear factor-κB (NF-κB) and pro-caspase-1, that leads to inflammasome formation (71–73). In addition, microglial stimulation by CgA led to iNOS-derived NO release at a comparable or even more effective level than bacterial LPS (74, 75). CgA induces a more neurotoxic phenotype in those cells, resulting in higher NO production and expression of certain neurotoxins such as FasL. This possibly leads to further neuronal degradation in certain diseases such as Alzheimer’s (51, 76).

CgA also plays a role in regulating endothelial and intestinal barrier function. In UC patients, CgA correlates negatively with the expression of tight junction proteins (such as occludin, zonula occludens-1 (ZO-1), and claudin) in colonic mucosal biopsies, disrupting the intestinal wall barrier (70). This effect on the intestinal epithelium, along with the mentioned earlier IL-8 and IL-18 expression enhancement, probably contributes to the progression of certain diseases like IBD (70).

Studies on human umbilical vein endothelial cells and microvascular endothelial cells (HMEC-1), as well as murine liver blood vessel permeability examination, show a rather anti-inflammatory effect of CgA, leading to decreased permeability of the endothelium (77–79). Administration of CgA in HMEC-1 cell cultures reduced the expression of ICAM-1 induced by TNF (77), acting synergistically with TNF-soluble receptors (78).

CgA is present in several immune-related conditions, as shown above. However, the direct effect of full-length CgA on inflammation is still a topic of debate. CgA is a precursor to many bioactive peptides. Therefore, its action in inflammation is likely to be caused by its derivatives, which are described in the next chapters. The direct action of full-length CgA cannot be excluded, though, since full-length CgA administration stimulates microglia and endothelial cells *in vitro*. Further studies are required to determine whether this effect is due to the full-length CgA protein or if it also results from CgA cleavage products present in the cell medium due to the activity of various proteases.

**Figure 2:**
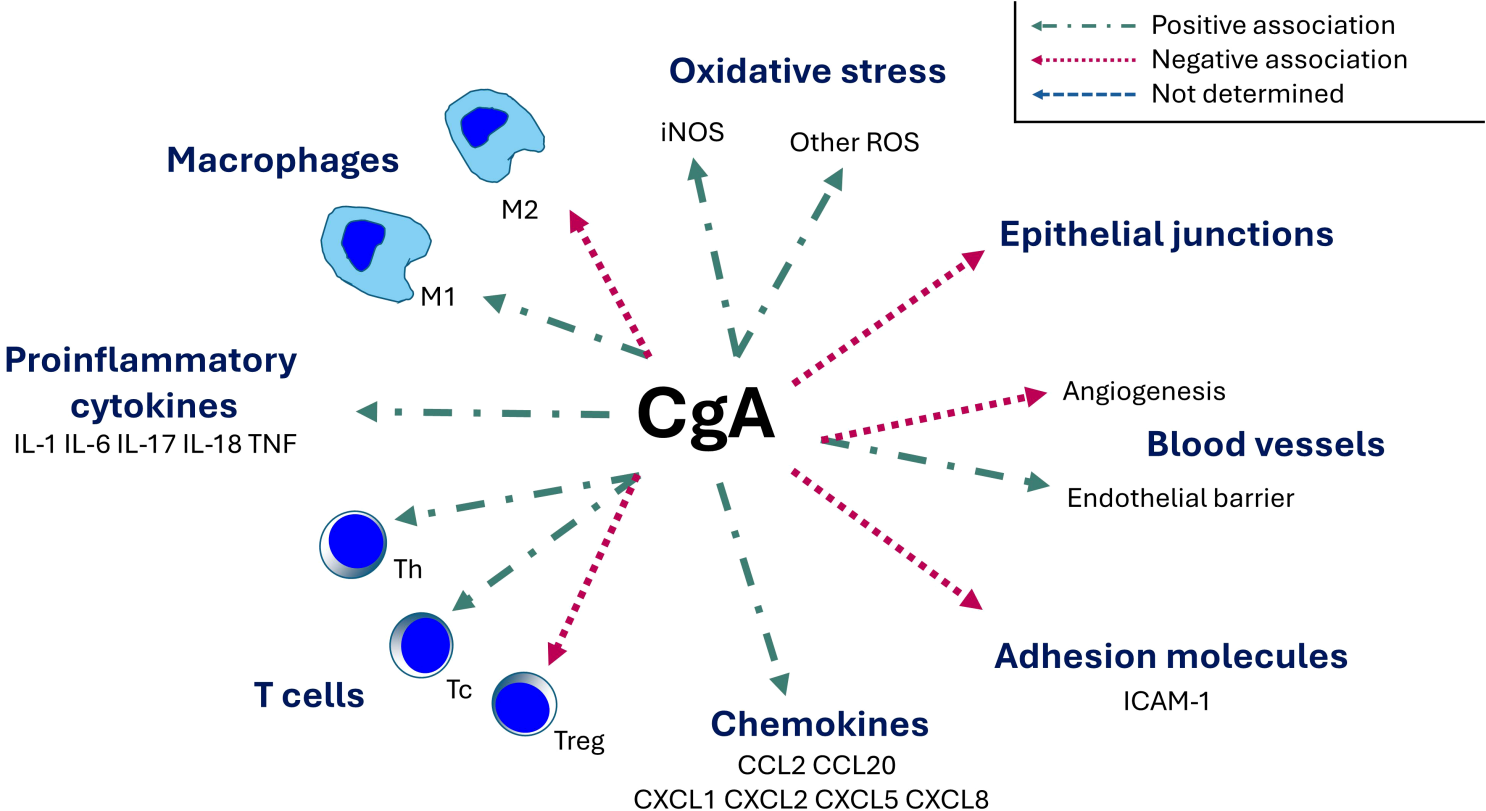
Immunomodulatory role of chromogranin A. CgA, chromogranin A; ICAM-1, intercellular adhesion molecule 1; IL, interleukin; iNOS, inducible nitric oxide synthase; M1, classically activated macrophage (proinflammatory); M2, alternatively-activated macrophage (anti-inflammatory); ROS, reactive oxygen species; Tc, cytotoxic T cell; Th, T helper cell; Treg, regulatory T cell; TNF, tumor necrosis factor.

The role of CgA in the modulation of inflammatory responses is summarized in **Figure 2**.

## 3 Catestatin

The vasoactive CgA-derived peptide CST was recognized for its ability to inhibit catecholamine release in PC12 cells and bovine chromaffin cells (29), human primary hippocampal neurons (80), and mouse adrenal medulla (81). Later studies recognized CST as a multifunctional peptide with many other biological functions (82, 83).

The role of CST in inflammation is multifaceted. Its effects against inflammation were shown in long-term inflammation, autoimmune diseases, and metabolic disorders. High CST levels were noticed in IBD, coronary heart disease, and after acute myocardial infarction (48, 84). On the other hand, lower plasma CST concentrations were found in patients with type 2 diabetes (T2DM) and high blood pressure (44, 85). Lower CST levels in these cases may be due to an inefficient cleavage of CgA (85).

CST’s ability to reduce catecholamine release and sympathetic overactivity leads to reduced blood pressure, as observed in CHGA gene knockout mice (86, 87) and CST knockout mice models (88). It has also been observed in polygenic hypertensive animal models such as BPH/2J mice (87) or spontaneously hypertensive rats (SHRs) (89). The antihypertensive action of CST could also be due to increased histamine release (90). It also regulates cardiac contractility, fostering general cardiovascular well-being (91). Furthermore, CST improves baroreflex sensitivity (92) and stabilizes heart rate variability (93), two mechanisms essential for cardiovascular homeostasis. Apart from its cardiovascular effects, CST drives angiogenesis, supporting new blood vessel development (19, 34). CST also supports metabolic health by stimulating lipolysis and beta-oxidation of fatty acids (94). Its protective roles extend to anti-atherogenic effects, reducing the risk of atherosclerosis (95), and anti-inflammatory properties, which mitigate systemic inflammation and tissue damage (44, 82).

CST shows many interactions with the cytokine signaling pathways, making this peptide an important regulator in the inflammatory process. Cytokines are important mediators of inflammation, which operate in a bidirectional manner with CgA and CST, influencing and being influenced by proteolytic processing and activity of these molecules. High levels of pro-inflammatory cytokines, such as IL-1β, IL-6, and TNF, have been traced in systemic and localized forms of inflammation, in which CST offers a protective counterbalance (82, 96–99). Studies in murine DSS-induced colitis models show that CST treatment significantly reduced colonic levels of pro-inflammatory cytokines such as IL-1β, IL-6, IL-18, and TNF. Mechanistically, these effects were linked to activation of STAT3 (97).

CST has been shown to modulate macrophage polarization decreasing M1 markers and promoting an anti-inflammatory milieu in CST knockout mice, as well as in DSS and 2,4 dinitrobenzene sulfonic acid-induced colitis models (44, 82, 88, 98). It also alleviated gut macrophage-mediated inflammation, measured by decreased levels of TNF and IL-1β (98, 99). This resulted in reduced immune cell infiltration (including macrophages and T cells) thus highlighting CST’s ability to act on the local immune microenvironment (98, 100).

In human studies, CST also showed an equivalent potential to reduce inflammation. CST suppressed TNF-induced expression of cytokines and adhesion molecules in endothelial cells through activation of ACE2 in patients with coronary artery disease (CAD) (96). In the ApoE−/− mice model such a response diminished leukocyte-endothelium interactions ex vivo and *in vivo*, suggesting the potential use of CST in preventing further immune-mediated damage to the endothelium and therefore to the progression of atherosclerosis (95, 96, 101).

CST also regulates immune cell chemotaxis and pro-inflammatory mediator production. In particular, CST-induced monocyte chemotaxis is mediated by G-protein-coupled receptor pathways and depends on PI3K and ERK signaling activation (102). On the contrary, however, CST may also inhibit chemotaxis towards pro-inflammatory CCL2 or CXCL2 chemokines and thus prevent immune cells from overactivation in inflammatory conditions (82, 103).

Degranulation of mast cells, an important process in acute inflammatory response, is another possible CST activity target. In an *in vitro* assay, CST has been found to trigger dose-dependently mast cell degranulation, leading to the secretion of histamine, eicosanoids (LTC4, PGD2, and PGE2), and chemokines (CCL2, CCL3, and CCL4), acting pro-inflammatory, opposing to the anti-inflammatory action of CST described above (50, 104).

CST may also modulate reactive oxygen species (ROS) production (105). In LPS-induced cardiomyocyte injury models, CST reduced oxidative damage by inhibiting the NF-κB, MAPK, and JNK signaling pathways. This resulted in decreasing ROS levels and protection from inflammatory-mediated tissue injury (106). Collectively, these results indicate the potential of CST to ameliorate oxidative stress and the release of inflammatory mediators seen in both acute and chronic inflammation.

The proper function of epithelial and endothelial barriers is critical to minimize inappropriate immune cell infiltration and to maintain tissue homeostasis. Previous studies have demonstrated that CST restores intestinal barrier integrity in models of IBD, where CST supplementation increased the expression of important tight junction proteins, including ZO-1 and occludin, in the colonic epithelium of DSS-treated mice and CST knockout mice (97, 100). In these models, CST also ameliorated colitis severity, as indicated by lower histological scores and reduced colonic permeability. *In vitro* data using DSS or LPS-treated Caco-2 epithelial cells supported this ability of CST to stabilize the epithelial barrier by showing increased cell viability, proliferation, and migration, along with rises in ZO-1 and claudin-1 levels with CST treatment (97).

The role of CST in protecting vascular endothelial integrity has also been reported. In TNF-treated endothelial cells, CST diminished the expression of adhesion molecules (e.g., P-selectin and E-selectin), thus also decreasing leukocyte adhesion and inflammation in the endothelium (96, 107). In murine acute pulmonary embolism, CST inhibited the TLR4-p38 signaling pathway, which attenuates endothelial inflammation as evidenced by reduced plasma levels of MCP-1 and myeloperoxidase (MPO) (107).

Taken together, these studies demonstrate CST’s ability to reduce levels of pro-inflammatory cytokines and ROS production. They also show CST’s ability to maintain the barrier function in both the gut and vascular systems by governing the integrity of tight junctions and suppressing immune cell infiltration. Interestingly, CST’s effect on mast cell degranulation and monocyte chemotaxis seems to be contradictory - rather pro-inflammatory. However, those pro-inflammatory actions are based on *in vitro* studies and are dose-dependent. Further studies are required to understand those processes and determine if this action of CST is also present *in vivo*.

**Figure 3:**
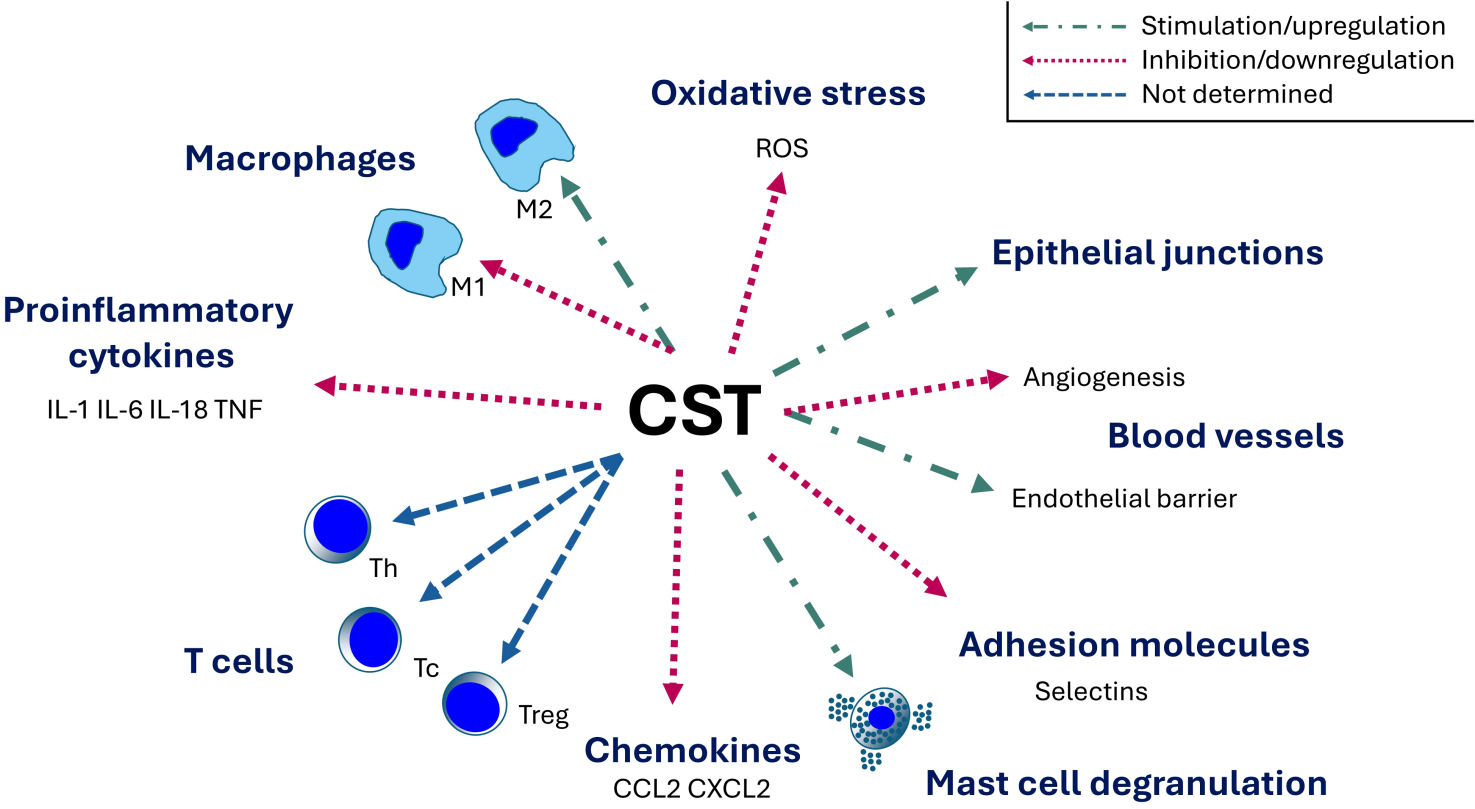
Immunomodulatory role of catestatin. CST, catestatin; IL, interleukin; iNOS, inducible nitric oxide synthase; M1, classically activated macrophage (proinflammatory); M2, alternatively-activated macrophage (anti-inflammatory); ROS, reactive oxygen species; Tc, cytotoxic T cell; Th, T helper cell; Treg, regulatory T cell; TNF, tumor necrosis factor.

The role of CST in the regulation of inflammatory responses is summarized in **Figure 3**.

## 4 Pancreastatin

PST was named after its initial discovery in the porcine pancreas and its ability to inhibit the secretion of insulin from pancreatic islets (28). It was the first CgA derivative detected in human plasma (50, 108). PST has a variety of forms with different molecular sizes, formed due to various proteolytic cleavage sites. All those forms share a common C-terminus, conserved between species (109).

PST is believed to have pro-inflammatory properties and has been implicated in inflammatory diseases, such as UC, or inflammation-related changes in T2DM (22, 110, 111). Elevated plasma PST was found in T2DM (41) and essential (primary) hypertension (112). Increased expression and PST levels in the colon of patients with UC are associated with higher pro-inflammatory cytokine expression (IL-8 and IL-18) and decreased expression of anti-inflammatory cytokines associated with alternatively activated macrophages (IL-10) (22). They are also associated with disruption of epithelial homeostasis due to reduced expression of tight junction proteins and suppression of anti-inflammatory macrophage differentiation (reduced mRNA markers of alternatively activated macrophages such as IL10, mannose receptor, CD1B were found) (22). The administration of the PST variant peptide PSTv1, which lacks PST activity, resulted in reduced inflammation and prevention of insulin resistance in high-fat diet (HFD) mice (43). Injection of insulin resistance inhibitor peptide-8 (PSTi8) in a murine model of T2DM protected against oxidative stress (110). This inhibitor also mitigated inflammation in a dexamethasone-induced fatty liver disease model via hepatic glucose release suppression, lipid accumulation reduction, and attenuation of oxidative stress. PSTi8 enhances insulin sensitivity while curbing inflammation associated with incomplete fatty acid oxidation through modulation of the AMPK signaling pathway (111).

PST may enhance inflammation by exerting various effects on the immune system. It increases the expression of certain pro-inflammatory cytokines (IL-1β, IL-6, IL-12, IL-18, TNF) and chemokines (IL-8 and MCP-1) in various tissues including intestinal tissue, white adipose tissue (WAT), pancreatic islets, and microglia (22, 43, 50, 109, 113). PST is also considered a microglial activator. It increases the protein level of IL-1β in hippocampal slice cultures (113). PST-treated hepatocellular carcinoma (HepG2) cells demonstrated increased mRNA expression of IL-1β, IL-6, and TNF compared to control cells (110). PST treatment of peritoneal macrophage cultures obtained from wild-type and CHGA gene knockout mice increased expression of IL-6, IL-12p40, MCP-1, and TNF mRNA (43). A positive correlation was observed between PST and the mRNA expression of IL-8 and IL-18 in the colon of patients with UC (22). Consistently, in mice with DSS-induced colitis, PST administration caused an increased colonic release of IL-18 (22). PST treatment also caused increased expression of IL-1β, IL-6, MCP-1, TNF, and iNOS in the adipose tissue of CHGA-knockout mice fed a HFD (43). In mice with elevated circulating PST levels due to chronic insulin administration, PST stimulated the epididymal WAT expression of IL-6, IL-12, and TNF genes and increased circulating levels of MCP-1 and TNF (114).

The inhibition of PST leads to a reduction of pro-inflammatory cytokine levels. In murine models of T2DM (db/db, mice fed a HFD, mice fed a high-fructose diet), the administration of PSTi8 caused a decrease in the plasma concentrations of IL-6 and MCP-1 (115). In another study on a murine model of T2DM (mice fed a HFD), PSTi8 reduced the liver tissue expression and serum levels of IL-1β, IL-6, and TNF, which were overexpressed in mice with uninhibited activity of PST (110). PST inhibition by PSTi8 reduced pro-inflammatory gene expression (IL-6, MCP-1, and TNF) in the epididymal WAT in a murine T2DM model (116).

Furthermore, PST decreases the expression of molecules such as IL-10 and Arginase-1 (Arg1) that are anti-inflammatory and favor tissue repair. The level of the PST protein in colonic tissue from patients with UC correlated negatively with IL-10 mRNA expression. A negative correlation was also observed between PST administration and IL-10 and Arg1 mRNA expression in mice with DSS-induced colitis (22). PST decreased the expression of Arg1 and IL-10 in the adipose tissue of CHGA-knockout mice fed a HFD (43). Consistently, inhibition of PST by PSTi8 led to an increased expression of Arg1 in the epididymal WAT of mice fed a HFD (117).

PST treatment of peritoneal macrophage cultures (obtained from wild-type and CHGA-knockout mice) increased the expression of mRNA for the mentioned previously pro-inflammatory: IL-1β, IL-6, IL-12p40, MCP-1, TNF, as well as iNOS (43). PST expression in the colon of patients with UC was also correlated negatively with the expression of M2 macrophages, which exert anti-inflammatory properties, further supporting the pro-inflammatory role of PST (22).

PST inhibition by PSTi8 led to increased macrophage polarization to anti-inflammatory M2 profile, attenuated inflammatory CD4+ T and CD8+ T cells, and increased the anti-inflammatory Treg cell and eosinophil populations in epididymal WAT in a murine T2DM model (116). Similarly, in another study, in the epididymal WAT of PSTi8-treated mice fed an HFD, an increase in anti-inflammatory cells (eosinophils, Treg cells, and M2 macrophages - F4/80+, CD206+) along with a reduction in pro-inflammatory cells (CD4+, CD8+ T cells and M1 macrophages - F4/80+, CD11c+) were observed in flow cytometry examination. The protein expression of iNOS and MHC-class II molecules was also reduced. Thus, the inhibition of PST reduces adipose tissue inflammation in the model of diet-induced obesity (117).

Moreover, PST enhanced ROS production in cultured 3T3L1 adipocyte cells and in the epididymal WAT of mice chronically treated with insulin (114). In a murine model of T2DM, several hepatic tissue abnormalities were detectable: disrupted cellular and mitochondrial ROS levels, changes in oxygen consumption rate and mitochondrial membrane potential, reduced ATP levels, and reduced NADP/NADPH ratio. Those aberrations coincided with the elevation of inflammatory markers (IL-1β, IL-6, and TNF) in tissues and serum. The administration of PSTi8 protected against those changes (110).

PST may also reduce the integrity of the colonic epithelium, thus promoting potential inflammation. In patients with UC, colonic expression of the PST peptide was correlated negatively with mRNA expression of tight junction proteins such as claudin, ZO1, E-cadherin, and occludin (22). Research on mice with intraperitoneal administration of PST also showed an effect opposite to this of CST on the intestinal barrier (100).

**Figure 4:**
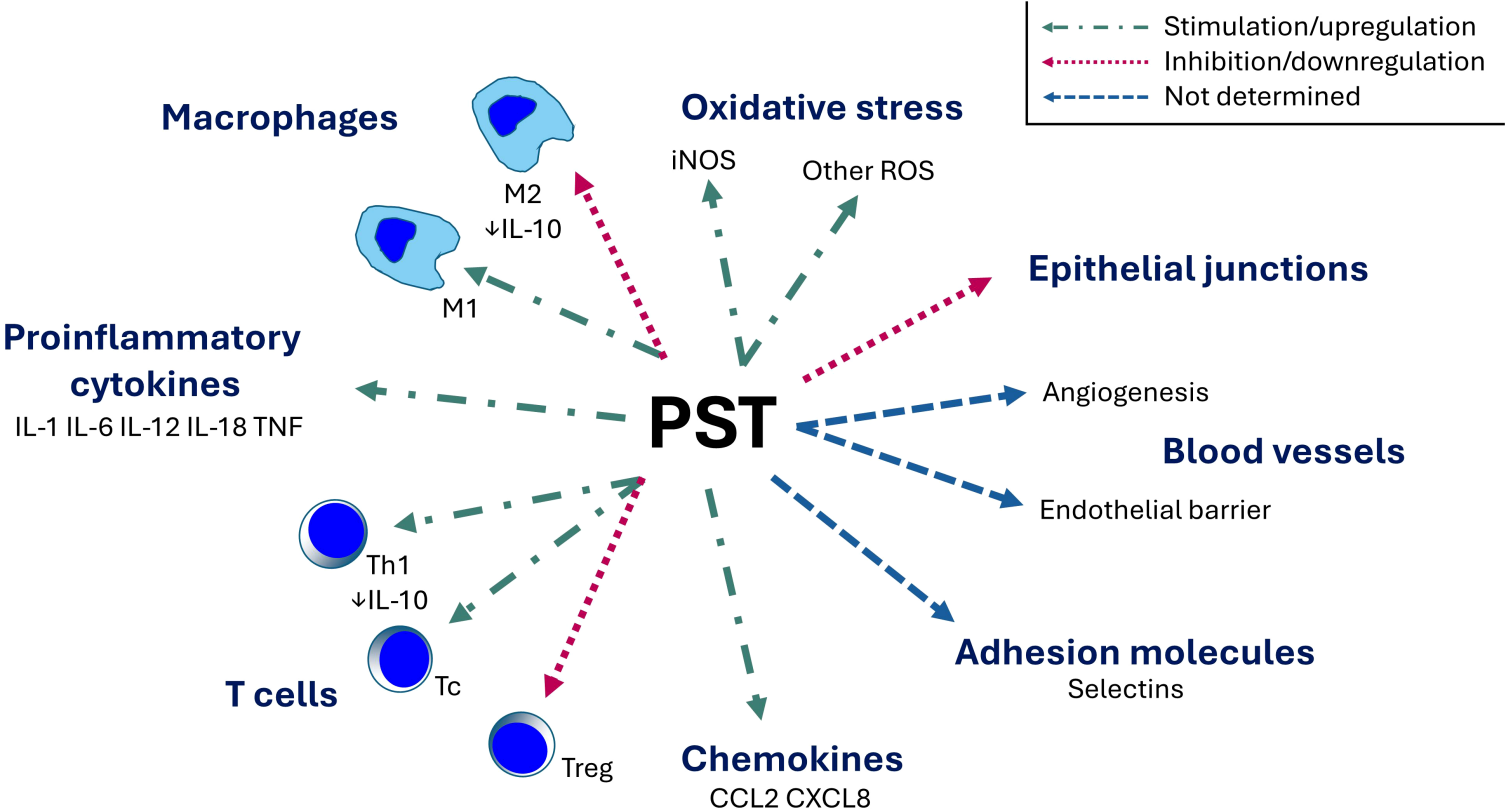
Immunomodulatory role of pancreastatin. PST, pancreastatin; IL, interleukin; iNOS, inducible nitric oxide synthase; M1, classically activated macrophage (proinflammatory); M2, alternatively-activated macrophage (anti-inflammatory); ROS, reactive oxygen species; Tc, cytotoxic T cell; Th, T helper cell; Treg, regulatory T cell; TNF, tumor necrosis factor.

As summarized in **Figure 4**, PST emerges as a peptide exerting pro-inflammatory effects. It may play an important role in various disorders associated with inflammation as it increases levels of proinflammatory cytokines, promotes proinflammatory M1 macrophage polarization, enhances ROS production, and reduces the intestinal barrier.

## 5 Vasostatin I, II and chromofungin

VS-I, VS-II, and CHR are biologically active peptides derived from the N-terminal fragment of CgA. Among these 3 peptides, VS-II is the longest one and contains the whole VS-I sequence. CHR, however, is an active 20-amino acid domain of both VS-I and VS-II (118, 119). The most highly studied peptide, VS-I, plays an anti-inflammatory role and is associated with several cardiovascular diseases (like atherosclerosis), CAD, RA, and IBD (40, 119–121). Increased VS-I blood levels were found in sepsis, ileal and pancreatic neuroendocrine neoplasms, Takayasu arteritis, and carotid atherosclerosis (120, 122, 123). VS-II contributes to various biological functions, especially those associated with the cardiovascular system, including vasodilation and atherosclerosis prevention (1, 124). VS-II levels were found to be significantly decreased in patients with CAD and patients with ischemic chronic heart failure (40, 125). CHR also exhibits anti-inflammatory properties that influence conditions such as CD, UC, sepsis-induced acute lung injury, atherogenesis, or CAD (126, 127).

### 5.1 Vasostatin I

As key regulators, VS peptides, in particular VS-I, exert complex effects on critical physiological functions, influencing vascular integrity, and angiogenesis in wound healing and inflammation, while also playing a pivotal role in tumor development (40, 48, 128).

In human cell culture assays, VS-I modulates and suppresses vascular adhesion molecule expression (such as VCAM-1, E-selectin) and reduces monocyte adhesion, and chemotaxis (via reduction of certain chemokines like MCP-1) to the endothelium (119, 129). It also reduces the activity of pro-inflammatory M1 macrophages, suppressing the secretion of cytokines like IL-6, resulting in a decreased inflammatory reaction (129). It is speculated that this anti-inflammatory effect of VS-I on the endothelium may be caused by a G inhibitory alpha protein that inhibits TNF-induced p38MAPK activation and thus regulates the responses of these cells to pro-inflammatory stimuli (130), preserving endothelial cell integrity in RA or e.g. heart failure (119).

It has also been shown that VS-I suppresses VEGF-induced migration, proliferation, and morphogenesis of the human umbilical vein endothelial cells. This implies that VS-I modalities not only stabilize the endothelial barrier but also restrain angiogenesis (131). In addition, VS-I disrupts the effects of pro-inflammatory mediators like angiotensin II, oxidized low-density lipoprotein (ox-LDL), and the previously mentioned TNF, which are key contributors to the development of atherosclerosis (79, 129).

VS-I was also found to be a critical mediator of intestinal inflammation. It inhibits DSS-induced colitis, leading to reduced weight loss, inhibition of colonic pro-inflammatory cytokines such as TNF; and maintenance of intestinal transmembrane resistance. VS-I limits IFN-γ- and TNF-induced permeability of the colonic epithelium. Moreover, VS-I decreases IL-8 release by LPS-stimulated epithelial cells (132). VS-I can also participate in the repair of damaged colonic epithelial cells, facilitate endothelial barrier crossing, and play a role in angiogenesis regulation. Given that, it can preserve gut barrier integrity in the enteric nervous system (50).

Despite the rather anti-inflammatory action of VS-I in the gut and blood vessels, its action on the microglia presents to be pro-inflammatory. This is due to iNOS activation, causing increased NO production in the brain, and the release of TNF and neurotoxic factors that lead to neuronal apoptosis via Fas receptors. In fact, the CgA action on microglia, described in previous chapters, appears to be mainly due to the VS-I domain (1, 74).

### 5.2 Vasostatin II

Also, VS-II exerts vascular anti-inflammatory functions. Lower expression of VS-II in atherosclerosis may be associated with the progression of this disease (23, 133). VS-II markedly lowers the levels of pro-inflammatory markers, including VCAM-1, TNF, MCP-1, and chemokine receptor-2 (CCR-2) in aortic tissues. Circulatory MCP-1 and TNF are also negatively correlated with VS-II levels (134). Studies on human vascular smooth muscle cells (VSMCs) additionally reveal that VS-II overexpression inhibits TNF, hsCRP, and IL-1β release, and VS-II downregulation promotes an opposite effect. Furthermore, VS-II modulates molecules that facilitate cellular adhesion. The overexpression of VS-II enhances the release of sICAM-1 and sVCAM-1, while their downregulation inhibits their secretion (133). The decrease in cytokines and adhesion molecules after VS-II treatment reduces leukocyte adhesion to the arterial wall and reduces the human monocyte/macrophage chemotaxis. It probably does not lead to anti-inflammatory M2 macrophage (Arg1 positive) polarization though. This corresponds with a 64% reduction in monocyte/macrophage content of atherosclerotic lesions after VS-II administration (134).

**Figure 5:**
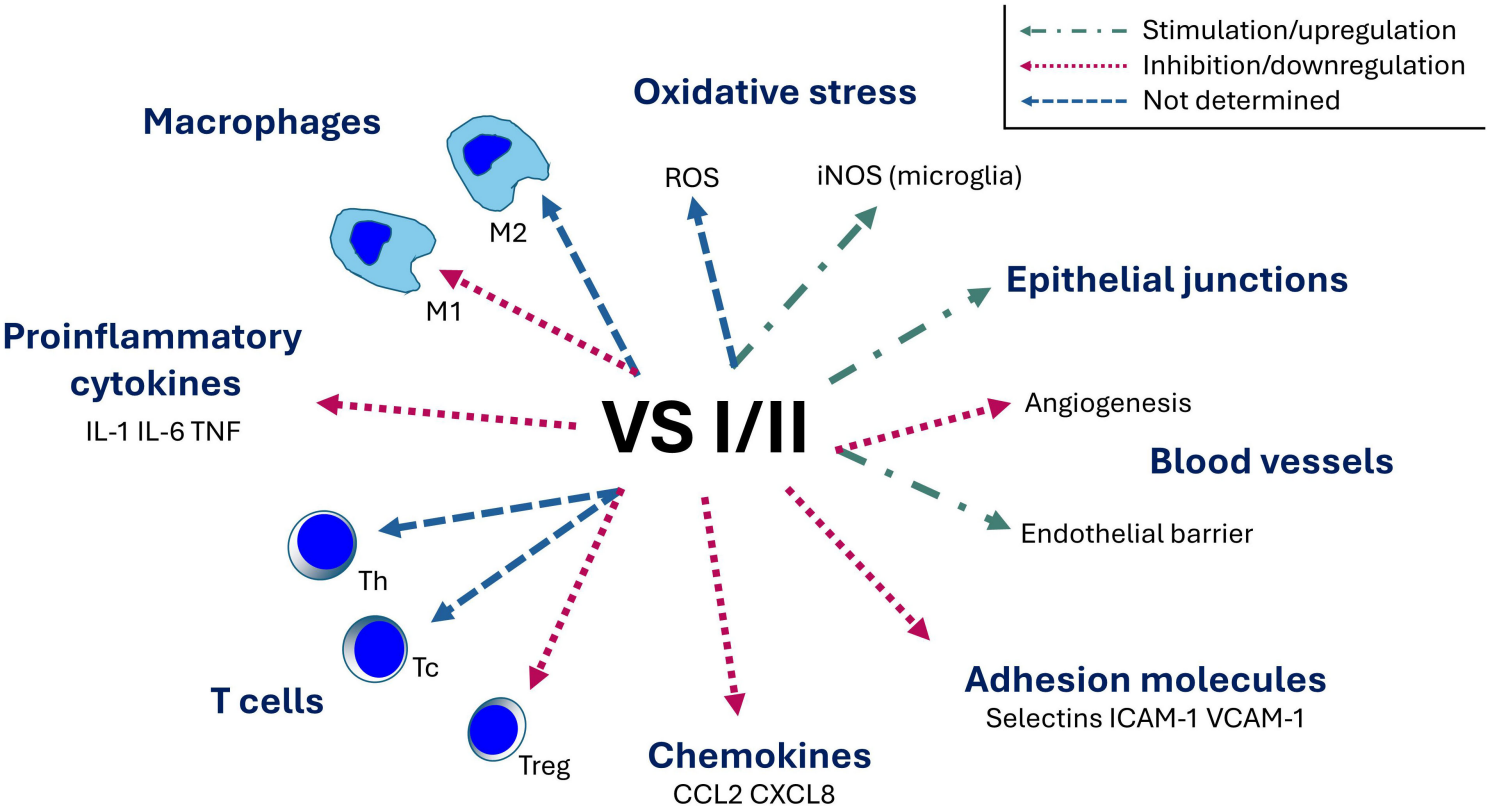
Immunomodulatory role of vasostatin I and II. VS I/II, vasostatin I/II; ICAM-1, intercellular adhesion molecule 1; IL, interleukin; iNOS, inducible nitric oxide synthase; M1, classically activated macrophage (proinflammatory); M2, alternatively-activated macrophage (anti-inflammatory); ROS, reactive oxygen species; Tc, cytotoxic T cell; Th, T helper cell; Treg, regulatory T cell; TNF, tumor necrosis factor; VCAM-1, vascular cell adhesion molecule 1.

The role of VS-I and II in the regulation of inflammatory responses is summarized in **Figure 5**.

### 5.3 Chromofungin

Being an active, short, 20-amino acid peptide, CHR emerges as a versatile modulator of inflammation through macrophage polarization via NF-κB downregulation and maintenance of epithelial, endothelial barrier (127, 135).

In active UC, colon tissue mRNA expression of CHR is downregulated when compared to healthy individuals (135). This downregulation negatively correlated with various important inflammatory markers such as pro-inflammatory macrophage (M1) cytokines (IL-1β, IL-6, TNF), colon IL-8 and IL-18, TLR−4 receptors, and NF-κB activity (135, 136). However, the expression of tight junction proteins and alternatively activated macrophage (M2) markers correlated positively with CHR levels (136). Notably, exogenous CHR administration in an animal DSS-colitis model caused a reduction of M1 macrophage markers and pNF-κB activity. It also seemed to reduce dendritic cell-related marker expression (such as CD11c, CD40, CD80, or CD86) in the colon, mesenteric lymph nodes, and spleen (126, 135).

Similar results were reported by studies on sepsis-induced acute lung injury. CHR seems to promote M2 macrophage polarization, and at the same time suppresses M1 polarization of alveolar macrophages, revealing a protective role against lung injury. It has been demonstrated that CHR inhibits the macrophage expression of NF-κB through the LBP/TLR4 signaling pathway. As a result, there is an elevated production of anti-inflammatory IL-4 and IL-10 along with repression of pro-inflammatory cytokines like IL-1β and TNF (137).

As mentioned, CHR increases intestinal tight junction protein synthesis, leading to decreased intestinal permeability. It also inhibits TNF-induced vascular permeability, preserving the endothelial barrier function. An *in vitro* assessment of cell permeability suggests that this effect can be at least partially explained because of the inhibition of Ca²^+^ influx to the endothelial cells through Store-Operated Calcium (SOC) channels. This mechanism prevents cytoskeleton rearrangement, reducing endothelial permeability and, as a result, could lead to e.g. reduced immune cell infiltration (127).

### 5.4 Summary

VS-I, II, and CHR have been highly studied in inflammatory processes in blood vessels and intestines, where they exert an anti-inflammatory effect, enhancing the endothelial and intestinal barrier, decreasing immune cell infiltration, and reducing pro-inflammatory markers. Interestingly, VS-I has been found to act pro-inflammatory in microglia, which means that the inflammatory signaling pathways can differ depending on the location. Further studies are required to recognize the signaling pathways of those peptides in inflammation. Additionally, studies are necessary to clarify the regulation and purpose of CgA cleavage to VS-I, VS-II, and CHR which share common sequences and seem to act synergistically in inflammation but have slight differences in effects, such as on M2 macrophage polarization.

## 6 Other CgA-derived peptides

Other less-known CgA derivatives may also play a role in inflammatory. Prochromacin exerts antimicrobial properties in *in vitro* bacterial cultures (27). Serpinin shows anti-apoptotic effect and reduces cellular death induced by oxidative stress in cell cultures (138). In an *in vitro* endocrine cell culture assay, it also inhibits the production of plasmin, which is present in the complex pathophysiology of colitis (30, 50). Therefore, it may potentially reduce plasmin-induced inflammation and decrease inflammatory oxidative stress (50). Little is known about the potential role of WE-14 in inflammation. Mast cell histamine release potentiation was reported after WE-14 stimuli. It also may act as an autoantigen which is discussed below (50, 139, 140).

Further studies are required to determine the exact role of these peptides in inflammation, as available data are very limited.

## 7 Chromogranin A as an autoantigen

Interestingly, CgA was also found to be an autoantigen able to trigger autoimmune responses. Anti-CgA autoantibodies can be found for example in non-small cell lung cancer patients; however, there is a strong bulk of evidence that CgA may play a role as an autoantigen in the pathogenesis of type 1 diabetes (T1DM) (141, 142).

In specific mouse models of diabetes, NOD (non-obese diabetic) mice with CHGA gene knockout did not develop diabetes. T cells reactive to CgA were found to be present in these mice but remained naïve. In NOD mice without any gene knockout, most of the T cells reactive to CgA were “antigen-experienced” (143). Lack of CgA-derived autoantigens in CHGA gene knockout NOD mice, however, may not be the only mechanism protecting from diabetes. Similar research showed that the absence of the secretory granule also impairs insulin antigen presentation and insulin formation and/or secretion from β-cells (144).

Certain CgA cleavage products have been found to act as autoantigens: WE-14, CgA 10–19, and CgA 43–52 (3, 145). The two latter peptides induce CD8+ T cell proliferation and cytotoxic activity while WE-14 is thought to activate both CD4+ and CD8+ diabetogenic T cell clones in *in vitro* binding and stimulation assays (141, 146). T-cell reactivity to WE-14 was found mainly in patients positive for the HLA-DQ8 allele (147). It is hypothesized that pancreatic β-cell-specific proteolytic processing of CgA along with certain post-translational modifications (such as transpeptidation and crosslinking) of CgA derivatives may be responsible for the specific autoimmune reaction to pancreatic insulin-secreting cells and not in other pancreatic cells or tissues (3, 141, 145).

The discovery of autoantigenicity of specific CgA-derived peptides: WE-14, CgA 10–19, and CgA 43–52, is an important step towards determining the exact role of CgA in T1DM. Further studies are required to understand the specific pancreatic β-cell processing of CgA and the degree of significance of those autoantigens in the complex etiopathogenesis of T1DM.

## 8 Anti-infectious and microbiota-modulatory properties of CgA and its derivatives

CgA also displays properties that directly affect microbes and fungi. Population-based studies have revealed strong correlations between CgA levels and microbial composition, as well as its diversity in the gut (50). CgA’s ability to negatively affect the growth of certain pathogenic/opportunistic bacteria may play a role e.g. in the complex pathophysiology of irritable bowel syndrome (IBS) (148). The antimicrobial and antifungal effects are achieved through CgA cleavage products, which are released by intestinal wall cells, certain immune cells – neutrophils, at the site of infection/injury, as well as systematically, e.g., by the adrenal medulla (1, 65, 149, 150). Plasma concentration of antimicrobial VS-I in patients undergoing coronary artery bypass surgery was found elevated just after surgical skin incision (150). Cationic structures of CgA derivatives, such as VS-I and its shorter active peptide, CHR, CST with its shorter antimicrobial peptide, cateslytin, and prochromacin can penetrate and destabilize the microbial/fungal membrane along with the fungal cell wall. Acting intracellularly, they possibly inhibit calcium/calmodulin-dependent enzymes (e.g., CaN phosphatases), suppressing DNA synthesis and interacting with organelles, causing induced cytoplasm vacuolization and mitochondrial damage (24, 82, 151–153). Furthermore, at the site of infection, CST, VS-I, and CHR also stimulate neutrophils to combat the infection in a positive feedback manner (50, 154).

Interestingly, CST was also found to inhibit the growth of the malarial parasite Plasmodium falciparum. Possibly, CST inactivates plasmepsins, aspartic proteases that degrade hemoglobin, thus blocking the parasite’s nourishment (91).

CgA cleavage products appear to be significant components of the innate immune system due to their broad-spectrum antimicrobial/antifungal/antimalarial activities and active immune-modulatory effects. Because of those properties, short, recombinant CgA peptides are studied as possible therapeutic agents during certain, difficult-to-treat, antibiotic-resistant infections (152, 155, 156).

## 9 Conclusion

**Figure 6:**
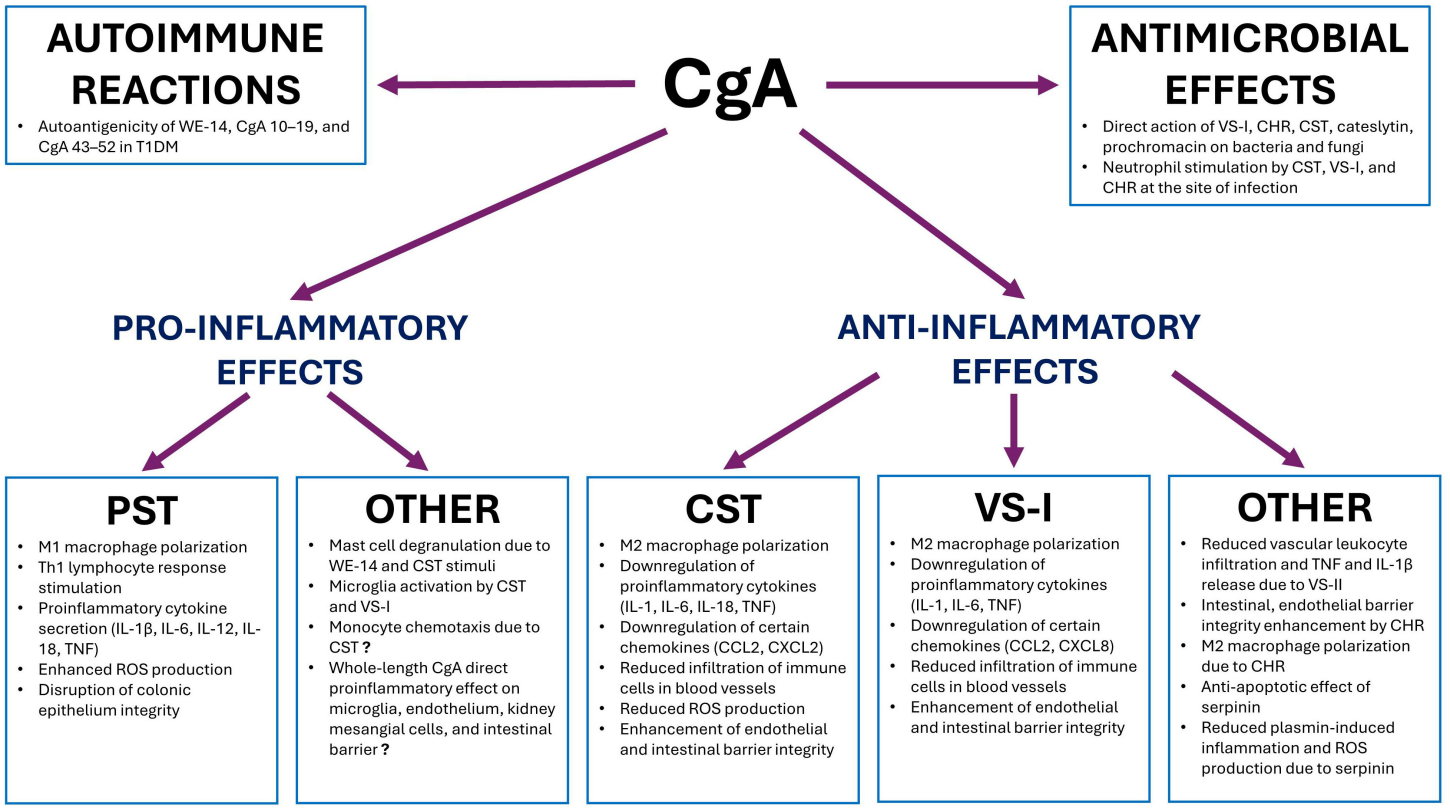
Role of chromogranin A and its main derivatives in inflammation, autoimmunity, and microbial infections – summary. CgA, chromogranin A; CHR, chromofungin; CST, catestatin; IL, interleukin; PST, pancreastatin; ROS, reactive oxygen species; T1DM, type 1 diabetes mellitus; TNF, tumor necrosis factor; VS I, vasostatin I.

CgA and its derivate peptides are elevated in certain autoimmune diseases such as IBD, CD, RA, SLE, or type 1 diabetes, as well as in a variety of other conditions also manifested by inflammation, thus suggesting their role in the pathogenesis of these disorders. Their role in inflammation, autoimmunity, and microbial infections is summarized in **Figure 6**.

It is difficult to evaluate the direct role of CgA in the regulation of immune reactions. The action of full-length CgA in inflammation cannot be fully excluded, but many of its activities can be attributed to its peptides. The positive correlation between CgA and intestinal pro-inflammatory markers, as well as its pro-inflammatory action on the intestinal barrier, could be explained by the activity of PST. Reduced permeability of the endothelium due to CgA can be explained by the action of VS-I and also CST. Finally, the activation of microglia by CgA can be attributed to the action of VS-I and PST.

CgA’s derivatives, especially CST, PST, VS-I, VS-II, and CHR, were found to affect various inflammatory mechanisms, sometimes acting synergistically, and sometimes antagonistically. Accordingly, it appears that PST plays a proinflammatory role via stimulation of M1 macrophage polarization and proinflammatory cytokine release (IL-1β, IL-6, IL-12, IL-18, TNF) as well as stimulation of Th1 responses. It also enhances ROS production and disrupts the integrity of the colonic epithelium.

On the contrary, CST and VS-I exert a rather opposing to PST, immunosuppressive effect, via downregulation of proinflammatory cytokines and stimulation of M2 macrophage polarization and Th2 responses. They also stabilize the endothelial and intestinal barrier integrity. Along with shorter CgA peptides like cateslytin, CHR, and prochromacin, CST, and VS-I have antimicrobial and antifungal properties, taking part in innate immunity. In addition, CST affects ROS levels, reducing oxidative damage. However, their exact effect on immune cells is not fully consistent, especially in terms of CST. They promote M2 macrophage polarization, decrease levels of certain chemokines such as CCL2, CXCL2, or CXCL8, and have been found to reduce infiltration of immune cells in blood vessels. On the other hand, they possess the ability to activate microglia and stimulate neutrophils at the site of infection. In *in vitro* assays, CST has also been found to induce monocyte migration and mast cell degranulation. Therefore, the exact effect of CST and VS-I on immune cells is probably dependent on the concentration of those peptides and an overall immunological/cytokine environment.

Other CgA derivatives also play roles in inflammation. CHR, like VS-I, enhances intestinal and endothelial barrier integrity and stimulates M2 macrophage polarization. VS-II seems to reduce vascular leukocyte infiltration and inhibits TNF and IL-1β release. It probably does not stimulate M2 polarization, though. Serpinin and WE-14 are also believed to possess a role in inflammation, but certainly further studies are required to determine it. In addition, certain derivatives - WE-14, CgA 10–19, and CgA 43–52 have been found to act as autoantigens, probably contributing to the development of T1DM.

The action of CgA and its derivatives strongly suggests that the final effect in various tissues and inflammatory models depends on the CgA cleavage pattern. Furthermore, it must be stressed that CgA-derived peptides may exert differential effects depending on the tissue or organs. Especially interesting is the pro-inflammatory, activatory effect of CST and VS-I on microglia, despite being overall rather anti-inflammatory. Taking everything together, CgA and CgA-derived peptides appear to be novel and interesting players in the field of immunity; however, their specific roles need further extensive clarification.
